# Frontal Contribution to Hippocampal Hyperactivity During Memory Encoding in Aging

**DOI:** 10.3389/fnmol.2019.00229

**Published:** 2019-09-25

**Authors:** Lars Nyberg, Micael Andersson, Anders Lundquist, Alireza Salami, Anders Wåhlin

**Affiliations:** ^1^Department of Radiation Sciences, Umeå University, Umeå, Sweden; ^2^Department of Integrative Medical Biology, Umeå University, Umeå, Sweden; ^3^Umeå Center for Functional Brain Imaging (UFBI), Umeå University, Umeå, Sweden; ^4^Department of Statistics, School of Business, Economics and Statistics, USBE Umeå University, Umeå, Sweden; ^5^Aging Research Center, Karolinska Institutet and Stockholm University, Solna, Sweden; ^6^Wallenberg Centre for Molecular Medicine, Umeå University, Umeå, Sweden

**Keywords:** hippocampus, pattern completion bias, aging, episodic memory, cognitive control

## Abstract

Hippocampal *hypo*- as well as *hyper*-activation have been reported during memory encoding in older individuals. Prefrontal cortex (PFC) provides top-down state signals to the hippocampus that bias its computation during memory encoding and retrieval, and disturbed top-down signals could contribute to hippocampal hyper-activation. Here, we used >500 cross-sectional and longitudinal observations from a face-name encoding-retrieval fMRI task to examine hippocampal hypo- and hyper-activation in aging. Age-related anterior hippocampal hypo-activation was observed during memory encoding. Next, older individuals who longitudinally dropped-out were compared with those who remained in the study. Older dropouts had lower memory performance and higher dementia risk, and hyper-activated right anterior and posterior hippocampus during memory encoding. During encoding, the dropouts also activated right prefrontal regions that instead were active during retrieval in younger and older remainers. Moreover, the dropouts showed altered frontal-hippocampal functional connectivity, notably elevated right PFC to anterior hippocampus (aHC) connectivity during encoding. In the context of a general pattern of age-related anterior hippocampal hypo-activation during encoding, these findings support a top-down contribution to paradoxically high anterior hippocampal activity in older dropouts who were at elevated risk of pathology.

## Introduction

The prefrontal cortex (PFC) is fundamental for cognitive control (Miller and Cohen, 2001). For episodic-memory processes, direct and indirect prefrontal-hippocampal anatomical connections have been highlighted (Simons and Spiers, 2003; Eichenbaum, 2017), and projections from the PFC have been shown to mediate top-down control of memory retrieval (e.g., Rajasethupathy et al., 2015; see also e.g., Kompus et al., 2011; Wais et al., 2018). The exact nature of fronto-hippocampal functional interactions is not known, but one possibility is that the PFC provides a state signal to the hippocampus that biases its computations to either pattern separation or completion processes, depending on goals and task instructions (e.g., whether episodic memories are to be encoded or retrieved). The two processes rely on differential but partially overlapping configurations of hippocampal circuitry; in pattern separation the entorhinal cortex conveys sensory signals to the dentate gyrus that performs an orthogonalization allowing a code with minimal overlap with previous representations to be projected to the CA3 subregion. In pattern completion, a sensory signal from the entorhinal cortex instead bypasses the dentate gyrus and is directly propagated to the CA3 subregion as a perceptual cue engaging an auto-associative network to recover a previously stored representation (Yassa and Stark, 2011). Thus, the same hippocampal subfield might be engaged in pattern separation during encoding and pattern completion during retrieval (Hunsaker and Kesner, 2013; Deuker et al., 2014), suggesting that external state signals may be required for flexible and voluntary shifts between modes of computations.

Conversely, altered prefrontal state signals and abnormal fronto-hippocampal connectivity may contribute to dysfunctional hippocampal processing, such as *hyper-activity* that has frequently been observed during memory encoding in aging, mild cognitive impairment, and in pre-symptomatic familial Alzheimer’s disease (Miller et al., 2008; O’Brien et al., 2010; Quiroz et al., 2010; Bakker et al., 2012). Such an elevated response may reflect a strengthening of the auto-associative network of the hippocampus CA3 subregion in aging, which may result in a shift in balance from pattern separation to pattern completion (Wilson et al., 2006). Additionally, age-related changes outside the hippocampal complex may contribute to hippocampal hyper-activation and a shift in balance from pattern separation to pattern completion (see Leal and Yassa, 2018). There is evidence for abnormal frontal functional responses (Miller et al., 2008; Browndyke et al., 2013) and fronto-hippocampal connectivity (Grady, 2012) in pathological aging, but whether disturbed frontal task-state signals contribute to hippocampal hyper-activation remains poorly understood.

Here, we tested the hypothesis that altered prefrontal state signals and abnormal fronto-hippocampal connectivity contribute to the hippocampal *hyper-activity*. We used an fMRI task that alternated between encoding and retrieval of face-name pairs (Salami et al., 2012; Pudas et al., 2018), administered within a longitudinal study that spanned over 20 years (Nilsson et al., 2004). Differential recruitment of right frontal regions during episodic-memory retrieval has been observed in previous cross-sectional analyses of this task (Salami et al., 2012; see Lepage et al., 2000; Habib et al., 2003), offering a way to decode hippocampal processes by analyzing distal patterns of frontal activity. We predicted that if hippocampal hyper-activity at encoding reflects improper state signals, possibly indicating the failure to shift between encoding and retrieval states, then hippocampal hyper-activity might be accompanied by elevated right-frontal cortex “retrieval signals” during encoding.

We considered both normal and putative pathological aging, by comparing older individuals who remained vs. dropped-out across two brain-imaging sessions administered 4 years apart (Pudas et al., 2018). Study-dropout at older age has been strongly associated with pathology, such as accelerated cognitive decline, dementia, and death (Sliwinski et al., 2003; Chatfield et al., 2005; Rabbitt et al., 2008; Glymour et al., 2012). Relatively few previous brain-imaging studies explicitly considered drop-out, despite evidence that the activation patterns for remainers vs. drop-outs can differ in significant ways (Nyberg et al., 2010; Rieckmann et al., 2017). Thus, while realizing that there may be many reasons for study dropout and that these may impact hippocampal processing in different ways, we considered dropout as a gross proxy for pathological aging. In line with previous claims (Maruszak and Thuret, 2014), we expected that hippocampal *and* frontal hyper-activity would be observed at the first imaging session for dropouts (i.e., in pathological aging), whereas longitudinal hippocampal hypo-activation would be seen from the first to the second imaging session for remainers (i.e., normal aging).

## Materials and Methods

### Participants

All participants in the present study were part of the Swedish *Betula* prospective cohort study on memory, health, and aging, and they were thoroughly characterized within that study including *APOE* genotyping (Nilsson et al., 2004). The research was approved by the local ethics board at Umeå University, and all participants provided written informed consent and were compensated monetarily for their participation. The participants included in this study were part of an imaging subsample of 376 participants, scanned in 2009–2010 (age range 25–80 years). In total, 53 subjects were not included in the baseline analyses due to pathology or missing/corrupt/incomplete data. The subjects were in some analyses aggregated into age groupings with the following mean age; 39 years, 59 years, 69 years, 77 years, and 81 years.

One-hundred and eighty-six participants returned for a follow-up scan approximately 4 years later. Of the 137 individuals who were not scanned at follow-up, 81 were classified as “true” dropouts and they formed the basis for the comparison of remainers and dropouts. The dropout rate (%) increased across the age groupings, with 40/10 (20%), 64/15 (19%), 61/23 (27%), and 21/33 (61%) remainers/true dropouts, respectively (i.e., the 81-year old group consisted of 21 remainers out of the 54 individuals who made up the 77 year-old grouping at the first imaging session, 4 years earlier). A minority (*N* = 56) of participants who were not scanned but participated in the health and cognitive examinations were not classified as “true” dropouts as they could not be scheduled for scanning within the time window allotted for the follow-up imaging session, resulting in *N* = 81 “true” dropouts.

Dementia diagnosis was done by a gero-psychiatrist as previously described (Mousavi et al., 2014; Boraxbekk et al., 2015).

### Offline Memory Testing

A composite score of five episodic memory measures (Nilsson et al., 2004) was utilized to quantify the participants’ objective memory performance. The composite included two tests of immediate free recall of sentences (16 items each; e.g., “lift the book”), two tests of category-cued recall of nouns from the sentences, and immediate free recall of a list of 12 unrelated nouns. The maximum composite score was 76 points. Test procedures remained constant across measurement occasions, but two different item-lists were alternated between test occasions to reduce practice effects. The composite score had a good level of internal consistency (Cronbach’s alpha: 0.83) and test-retest reliability (*r* = 0.79; Pearson correlation). The participants also provided a subjective estimation of longitudinal memory decline/improvement by answering the question “How do you think your memory is functioning today compared to 5 years ago?” They responded by selecting one out of five responses (1 = much worse; 2 = somewhat worse; 3 = same; 4 = somewhat better, 5 = much better).

### Episodic Memory fMRI Face-Name Task

The scanner task at both baseline and follow-up MRI was a face-name paired-associates task, described in detail in our previous work (Salami et al., 2012; Pudas et al., 2018). This 10-min task comprised six blocks of face-name encoding (remember a name associated with a face), six blocks of cued-name retrieval (indicate the first letter corresponding to the name previously encoded with a face), and eight blocks of an active control task involving a simple perceptual discrimination (pressing a button each time a fixation mark changed into a circle). Scanner task performance was calculated as mean number (%) of correct answers. Mean duration between encoding and retrieval of a given face was 85.1 s (SD = 26.1 s). Block order was pseudo-randomized and constant across participants. Each block comprised four items, which were color photographs of unfamiliar faces, presented for 4 s each. Responses were given through a button press on a scanner-compatible response pad, and participants were instructed to guess if uncertain. All participants completed a short practice version of the task at least once prior to scanning. In the scanner room, the task was displayed on a computer screen seen through a tilted mirror on the head coil.

### fMRI Acquisition

The same 3T General Electric scanner (equipped with a 32-channel head coil) was used to collect images at both imaging sessions. Functional images were acquired with a gradient echoplanar imaging sequence [37 transaxial slices; thickness: 3.4 mm, gap: 0.5 mm, repetition time (TR): 2,000 ms, echo time (TE): 30 ms, flip angle: 80°, field of view: 25 × 25 cm, matrix: 96 × 96 voxels (zero-filled to 128 × 128)]. Ten dummy scans were collected and discarded prior to experimental image acquisition to allow for progressive saturation of the signal. Subject head movement was minimized using cushions inside the head coil. The scanner underwent standard maintenance and upgrades during the interval between the baseline and follow-up scans of this study. A quality assurance routine was carried out on a weekly basis since November 2010 to assure signal stability, and the recording indicated satisfactory within-scan scanner stability (Pudas et al., 2018).

### Preprocessing of Functional MRI Data

Functional data from both baseline and follow-up were preprocessed using SPM12 (Wellcome Trust Centre for Neuroimaging, Functional Imaging Laboratory^1^), implemented in MATLAB R2014b (MathWorks). The details of data analysis were reported in our previous work (Pudas et al., 2018). First, all images were corrected for differences in acquisition time (slice timing). Second, head movement corrections was carried out using the realign and unwarp function, by which each volume was rigidly aligned to the first volume of the series. Thereafter, realigned images were spatially normalized into a common space in a multi-step procedure employing DARTEL. This involved co-registering the individual’s functional images to the structural T1-image. Separate co-registrations were performed on data from baseline and follow-up MRI sessions, segmenting each individual’s structural T1-image into gray-matter, white-matter and cerebrospinal fluid components. Thereafter, DARTEL was used to create a template image of baseline and follow-up data for each participant, and these individual template images were subsequently merged into a group-level DARTEL template. The composite of subject-specific and group flow fields from these transformations were applied to the functional images to transfer them into template space. The images were finally affine aligned to Montreal Neurological Institute (MNI) space (using the default TPM MNI template), resliced to 2 × 2 × 2 mm, and smoothed with an isotropic 8 mm FWHM Gaussian kernel.

### Dynamic Causal Modeling

We used a Dynamic Causal Model (DCM), implemented as in a previous study (Büchel and Friston, 1997), with three regions in the right hemisphere, the fusiform face area (FFA), anterior hippocampus (aHC), and ventrolateral PFC (VLPFC). The regions were defined by 3 mm spheres around coordinates of peak activations retrieved from the main fMRI analyses, (*x*, *y*, *z*)—coordinates (38, −54, −20) for FFA, (22, −8, 16) for aHC and (34, 22, −4) for VLPFC, respectively. In DCM, an input region is required to perturb the system in question, and this region should be consistently activated during task conditions. As could be expected from using a face-name associative memory task, the FFA was previously found to be consistently activated during both states (encoding and retrieval vs. baseline; Salami et al., 2012) and was therefore selected as input region in here on basis of contrasts between encoding-baseline and retrieval-baseline. The connections in the DCM model were specified as follows: (i) bidirectional connections between FFA-aHC and aHC-VLPFC, respectively (the DCM A-matrix); (ii) input during both encoding and retrieval (the DCM C-matrix) to FFA; and (iii) allowing for modulation of the connection from VLPFC to aHC (the B-matrix) during both encoding and retrieval. Critically, the main analysis reported in the article concerns the degree of modulation of the task conditions on the VLPFC to aHC connectivity. Note, that we modeled aHC-VLPFC interactions as a direct link although this pathway likely also includes indirect polysynaptic routes. Default options in SPM12 were used to estimate the DCM for each subject during encoding and retrieval.

### Statistical Analyses

The analyses were implemented in SPM12. The preprocessed functional data were high-pass filtered (128 s), and voxel-wise general linear models were set up for each subject, with the experimental conditions from the scanner task (encoding, retrieval, and control) as regressors. Each regressor was modeled as a boxcar, convolved with the standard hemodynamic response function. In addition, six realignment parameters from the motion correction step of the preprocessing were included as covariates of no interest. Separate analyses were set up for baseline and follow-up fMRI data. Thereafter, subject-level contrast images were generated, comparing the experimental conditions of the scanner task, encoding vs. control, and retrieval vs. control. These contrast images were then carried on to random-effects group analyses, which proceeded in several steps. To identify hippocampal and frontal regions more activated during face-name encoding and retrieval relative to the control task, group analyses were performed as one sample *t*-tests of all subjects at baseline. We also investigated the reproducibility of the observed peaks by analyzing the follow-up data separately. Observed hippocampus peaks were labeled as either falling into the anterior or posterior hippocampus depending on their location relative to *y* = −21 mm in MNI space (Poppenk et al., 2013; Salami et al., 2016). To detect potential differences in activation between older remainers and older dropouts at baseline, a 2-by-2 RM-ANOVA was conducted in which the factors were condition (encoding and retrieval) and group (older remainers and older dropouts). Individual-level contrast values were compared across two groups (older remainer vs. older dropout) using one-sided, two-sample *t*-tests. For batching analyses and visualization of results, an in-house developed software (DataZ) was used. Bold change was calculated from the ratio of the contrast value and the constant obtained from the same voxel (the ratio was multiplied by 100 to get the expression in percent). All fMRI bar graphs were based on the peak voxel values. Visualizations of fMRI results on an inflated cortical surface was obtained with BrainNet (Xia et al., 2013).

Changes in anterior and posterior hippocampus peak activations, identified from whole brain analysis across the baseline sample, was modeled using separate Linear Mixed Effects models for the left and right side, respectively. Age was treated as a fixed effect, and a random intercept was used to account for correlated within-subject observations. We chose to separately analyze anterior and posterior hippocampal regions in view of meta-analytic evidence that the aHC is more strongly engaged during encoding and the posterior hippocampus during retrieval (Kim, 2015).

For functional connectivity as well as the DCM VLPFC to aHC modulation, Wilcoxon signed-rank-tests were used to investigate whether the connectivity was different between conditions, and secondly if there was a significant top-down VLPFC to aHC influence in encoding and retrieval, respectively. Mann-Whitney *U* tests were used to test differences in DCM derived top-down VLPFC to aHC modulation between groups.

To further investigate the direction of the connectivity between VLPFC and aHC, lag analyses were implemented by calculating the cross-correlation function between the investigated time-courses (Mitra and Raichle, 2018). Prior to calculation of time-courses, a voxel-level nuisance regression was performed to remove confounding signal variance. This regression removed global, white, CSF signals, and their derivatives, as well as 24 motion parameters (translation and rotation for current and previous frame and their squared versions). A delay with sub-TR resolution was obtained by interpolating the cross-correlation function using cubic splines. All lag statistics were based on non-parametric Wilcoxon signed-rank tests.

## Results

### Hippocampus and Prefrontal Activity During Encoding and Retrieval

**Figure 1:**
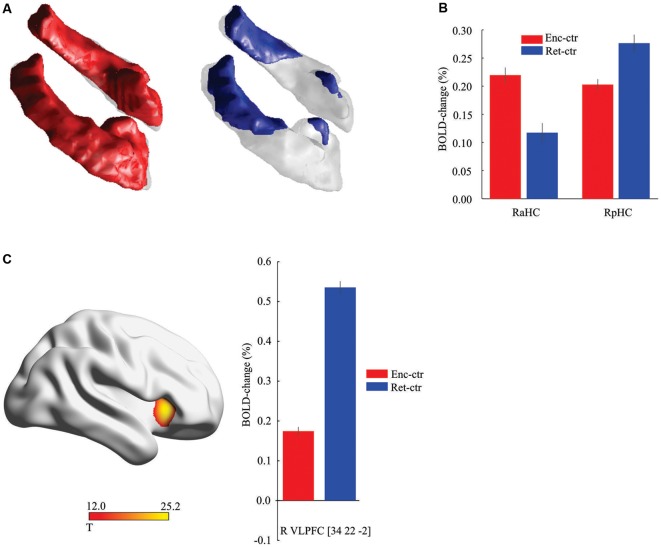
Hippocampal and frontal activation during encoding and retrieval. **(A)** Activation along the hippocampus longitudinal axis during encoding (red) and retrieval (blue) relative to control task (threshold, *t* > 8.0 for illustration). **(B)** Activity in the right anterior hippocampus (aHC) was stronger during encoding (*P* < 0.001, *t*_(322)_ = 6.51), and in the right posterior hippocampus (pHC) at retrieval (*P* < 0.001, *t*_(322)_ = 4.76). Data displayed from the peak voxels, collapsed across imaging sessions. **(C)**. Differential recruitment of right frontal cortex during retrieval. Data displayed from the peak voxel, collapsed across imaging sessions. Ctr, control task. Vertical lines = ± Standard Error of Mean (SEM).

The data from the baseline imaging session (*N* = 323) were first analyzed to identify hippocampal regions that were more activated during face-name encoding and retrieval relative to the control task (*p* < 0.05, FWE corrected). This analysis identified bilateral anterior (*x*, *y*, *z* = 22, −8, −16; *x*, *y*, *z* = −20, −10, −16) activation peaks during encoding (right *t*_(322)_ = 16.47; left *t*_(322)_ = 16.24) and retrieval (right *t*_(322)_ = 7.24; left *t*_(322)_ = 6.94), and also bilateral posterior (*x*, *y*, *z* = 26, −30, −2; left *x*, *y*, *z* = −24, −30, −4) activation peaks during encoding (right *t*_(322)_ = 22.29; left *t*_(322)_ = 21.53) and retrieval (right *t*_(322)_ = 19.05; left *t*_(322)_ = 19.78; Figure 1A). There was a high degree of stability in the activation pattern across imaging sessions (Dice coefficient > 0.80 at *p* < 0.05 FWE corrected), with significant activation changes in the same hippocampal regions also at the follow-up session for both encoding (right anterior *t*_(185)_ = 12.23; left anterior *t*_(185)_ = 12.70; right posterior *t*_(185)_ = 16.25; left posterior *t*_(185)_ = 16.70) and retrieval (right anterior *t*_(185)_ = 6.09; left anterior *t*_(185)_ = 6.38; right posterior *t*_(185)_ = 18.64; left posterior *t*_(185)_ = 19.37]. Thus, anterior and posterior hippocampus were recruited during both encoding and retrieval, although a plot of responses confirmed previous findings (Kim, 2015) of greater encoding- than retrieval activity in the aHC along with greater retrieval- than encoding-related activity in the posterior hippocampus (Figure 1B). Consistent with prior findings (see Habib et al., 2003), encoding-retrieval differences were also observed in cortical regions. In line with our prediction and prior studies (see “Introduction” section), here we focused on the right VLPFC (*x*, *y*, *z* = 34, 22, −2) that was more strongly activated at retrieval than at encoding (Figure 1C) at both the baseline (*t*_(322)_ = 18.90) and follow-up (*t*_(185)_ = 16.98) sessions.

### Hippocampal Hypo- and Hyper-Activation

**Figure 2:**
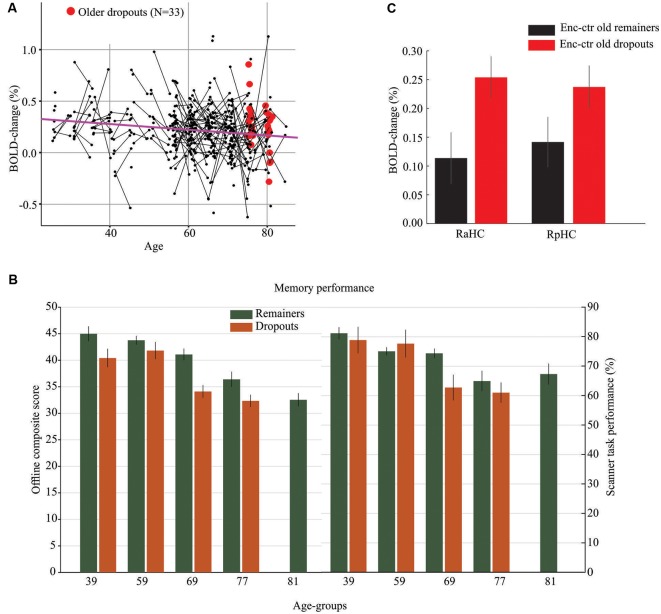
Hippocampal hypo- and hyper-activation in normal and pathological aging. **(A)** Age-related hypo-activation of right aHC during memory encoding. The purple line is the slope from a Linear Mixed Effect model and reflects an age-related decrease in hippocampus activity (hypo-activation). The black dots represent subjects with line connecting baseline and follow-up scans. Red dots denote older dropouts. **(B)** Offline and in-scanner episodic-memory performance for participants who remained in both sessions vs. dropped out after the first session. The oldest age group (*M* = 81 years) included only longitudinal observations for remainers from the first imaging session. **(C)** The right anterior (RaHC) and posterior (RpHC) hippocampus was hyper-activated at encoding for older dropouts relative to older remainers. Vertical lines = ± SEM.

The cross-sectional and longitudinal observations (*N* = 509, 323 scans from baseline and 186 scans from follow-up) were first used to examine age-related changes in anterior and posterior hippocampus activity at encoding and retrieval. Significant hypo-activation was observed in right (slope = −2.78*10–3, *F*_(1,305)_ = 10.74, *p* = 0.0012) and left (slope = −2.79*10–3, *F*_(1,305)_ = 9.70, *p* = 0.002) aHC during encoding (as illustrated for right aHC in Figure 2A), but not during retrieval (*p*’s > 0.05). No significant hypo-activation was found in the posterior hippocampus (*p*’s > 0.05).

Longitudinal analyses of age-related hippocampus hyper-activity during encoding and retrieval yielded no significant effects in the full sample (*p*’s > 0.05). To specifically examine hippocampal hyper-activity in what might represent pathological aging, we compared older dropouts with older remainers of the same age. Specifically, of the 54 individuals in the oldest age group at the baseline session (Mean age = 77 years), 21 returned for the second imaging session (Mean age of remainers = 81 years) whereas 33 only participated in the first session and dropped out from the follow-up. The two groups were compared on select demographic, clinical, and cognitive variables at baseline, and differences were observed for variables previously associated with study-dropout at older age (Table 1; Figure 2B). That is, the older dropouts displayed lower baseline offline episodic-memory performance and included a higher percentage of APOE-ε4 carriers. In addition, underscoring the pathological nature of study dropout, by the time for the follow-up imaging session, several individuals in the dropout group had progressed to dementia and death. A direct comparison of peak activations from the first scanning session for older dropouts and older remainers revealed significant hyper-activity for dropouts in the right aHC at encoding (*t*_(52)_ = 2.42, *p* = 0.01; Figure 2C; highlighted in red in Figure 2A). There was a similar trend in the right posterior hippocampus (*t*_(52)_ = 1.64, *p* = 0.03 Figure 2C). No significant hyper-activity was observed during retrieval in anterior or posterior hippocampus (*p*’s > 0.10).

**Table 1:** Demographic data, memory performance, and selected clinical data of the older remainer and older dropout groups at baseline.

	Older remainer	Older dropout
N	21	33
Age (range)	75–81 years	75–81 years
Sex	9 F/12 M	19 F/14 M
ApoE-ε4 carriers*	16%	45%
Offline episodic memory**	36 ± 7	32 ± 8
Subjective memory decline	76%	67%
Dementia^†^	0%	24%
Death^†^	0%	9%

*p = 0.033 according to a chi-square test. **p = 0.034 according to a *t*-test. ^†^over the next 4-year period.

### Frontal Hyper-Activity

**Figure 3:**
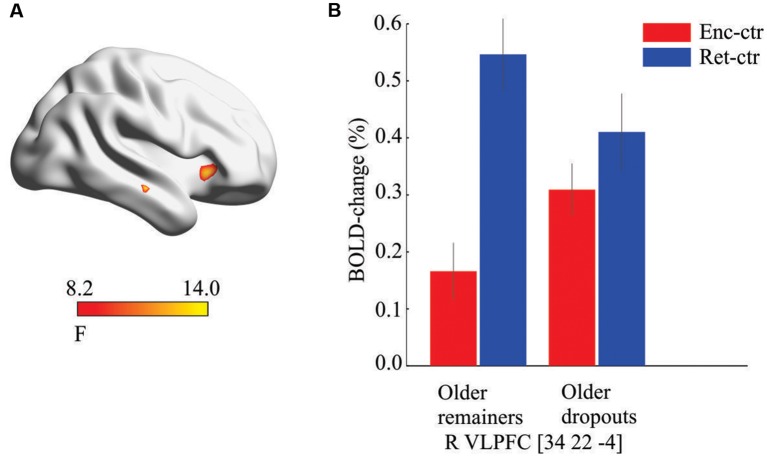
Prefrontal hyper-activation. **(A)** Significant interaction effect suggesting group-by-condition differences in the recruitment of ventrolateral prefrontal cortex (VLPFC) at baseline. **(B)** *Post hoc* analyses revealed that the VLPFC was differentially recruited during retrieval for older remainers but recruited to a similar degree during encoding and retrieval for older dropouts (bars derived from the interaction peak voxel, enc, encoding; ctr, control task).

To test the prediction of altered top-down signals during encoding, a 2 (older dropouts vs. older remainers) by 2 (encoding vs. retrieval) whole-brain ANOVA was conducted. A significant interaction effect was observed in right VLPFC (*x*, *y*, *z* = 34, 22, −4; *F*_(1,104)_ = 13.08; *p* < 0.001; *k* = 21, Figure 3A). This peak overlapped with the right prefrontal region that in the overall sample was differentially recruited at retrieval (Figure 1C). As in the overall sample, the older remainers recruited this region more strongly at retrieval than encoding (*t*_(20)_ = 6.70, *p* < 0.001), whereas it was recruited to a similar degree (*p* > 0.05) during encoding and retrieval by older dropouts (Figure 3B). A *post hoc* test confirmed right VLPFC hyper-activation at encoding for older dropouts compared to older remainers (*t*_(52)_ = 2.05, *p* = 0.01).

### Altered Fronto-Hippocampal Connectivity

**Figure 4:**
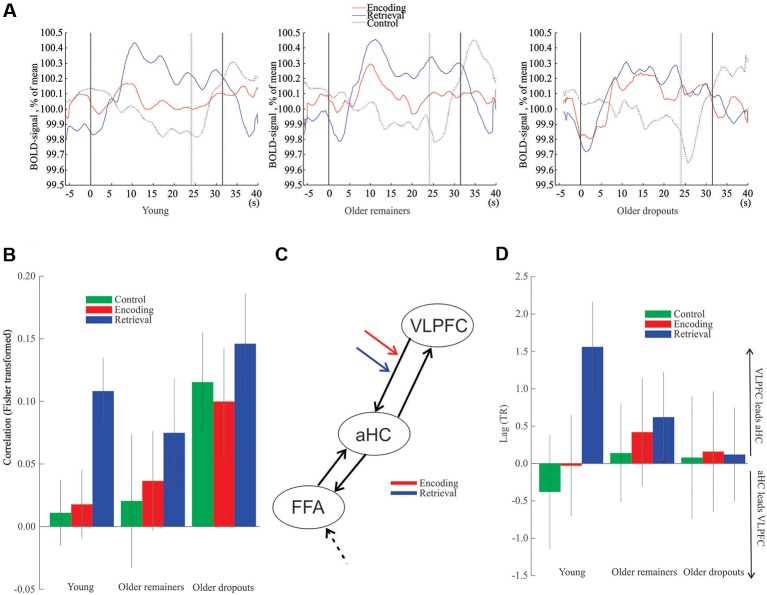
Hippocampus-frontal interactions. **(A)** Time-course data from right VLPFC peak voxel. The first solid vertical line represents task onset for encoding, retrieval and control blocks, respectively. The second solid vertical line represents the ending of encoding and retrieval tasks, and the dotted line represents ending of the control task. **(B)** Functional connectivity between right aHC and right VLPFC. **(C)** Structure of the dynamic causal model (DCM). The encoding and retrieval notations indicate the examined connection. The dashed black arrow to FFA represents input to the model. **(D)** Lag between the right aHC and the right VLPFC. Vertical lines = ± SEM. VLPFC, ventrolateral prefrontal cortex; aHC, anterior hippocampus; FFA, fusiform face area.

Finally, prefrontal-hippocampal functional connectivity was examined. Plots of the average BOLD signal during encoding and retrieval (Figure 4A) were used to illustrate the time-courses underlying the observation that right VLPFC (*x*, *y*, *z* = 34, 22, −4) was differentially recruited throughout the retrieval task for the older remainers, whereas for older dropouts the frontal signal was elevated to a similar degree during both encoding and retrieval. In addition, a young group (*N* = 50; Mean age = 39 years) expressed differential recruitment of right VLPFC during retrieval, comparable to that of the older remainers.

Analyses of functional connectivity of the right VLPFC (*x*, *y*, *z* = 34, 22, −4) with the right aHC (*x*, *y*, *z* = 22, −8, −16) demonstrated significant connectivity during retrieval for the young (*z*_(49)_ = 3.48, *p* < 0.001) and older remainers (*z*_(20)_ = 1.82, *p* = 0.034) but no significant connectivity during encoding or the control task (*p*’s > 0.05; Figure 4B). In contrast, for the older dropouts the right PFC showed aberrant functional connectivity with the hippocampus during all conditions including the control task (*p*’s < 0.001, Figure 4B).

Consistent with a top-down influence of right VLPFC to aHC during retrieval, a DCM analysis (Figure 4C) revealed a significant difference in modulation of the right VLPFC to aHC connection between encoding and retrieval for young subjects (*z*_(49)_ = 2.0, *p* = 0.045) and older remainers (*z*_(20)_ = 2.3, *p* = 0.022), but no difference was seen for older dropouts (*z*_(32)_ = 0.23, *p* = 0.82). Additional analyses confirmed that the basis for the non-significant difference for the older dropouts was significant VLPFC to aHC modulation during *both* encoding and retrieval (*z*_(32)_ = 2.42, *p* = 0.015 and *z*_(32)_ = 2.05, *p* = 0.034, respectively). Finally, direct comparisons of older dropouts with older remainers revealed significant differences in the VLPFC to aHC modulation during encoding (*z*_(52)_ = 2.86, *p* = 0.0043) but not during retrieval (*z*_(52)_ = 1.30, *p* = 0.19).

Finally, a lagged correlation analysis (Mitra and Raichle, 2018) indicated that right VLPFC activity preceded right aHC activity during retrieval for young (*z*_(49)_ = 2.79, *p* = 0.003) and old remainers (*z*_(20)_ = 2.05, *p* = 0.020) but not in any condition for older dropouts (*p*’s > 0.05; Figure 4D).

## Discussion

Large-scale longitudinal support was obtained for age-related anterior hippocampal hypo-activation during memory encoding. No significant hypo-activation was observed in anterior or posterior hippocampus during retrieval. Also, no hypo-activation during encoding was seen in the posterior hippocampus, which supports and extends previous cross-sectional findings (Ta et al., 2012). Thus, age-related hypo-activation of the hippocampus was region (anterior) and process (encoding) selective. Intriguingly, we also observed “hyper-activation” of the same right aHC region during encoding but not retrieval. Increased hippocampal activity was not seen across the full sample, but rather in the comparison of older dropouts with older remainers. This selectivity suggests that the elevated hippocampal response is not a feature of normal aging, but rather a sign of pathological aging such as minor neurocognitive disorder (Sachdev et al., 2014). Indeed, it has been suggested that hippocampal hyper-activation might serve as a biomarker for Alzheimer’s disease (Maruszak and Thuret, 2014).

Specifically, we found that the older dropout group showed reduced offline episodic memory, had a higher proportion of APOE-ε4 carriers, and included several who progressed to manifest dementia over the next 4-year period. Thus, the individuals in the dropout group should have been at elevated risk for pathological aging, if not already in the early stages of pathology at baseline. Here, it should be stressed that the limited size of our older dropout group prevented more refined analyses of whether hyper-activity was selective or differentially stronger for dropout individuals who converted to dementia, or whether it is equally expressed in diverse age-related pathologies (see Small et al., 2011). In future studies, when a greater number of individuals in the Betula study have acquired specific pathologies, more refined analyses will be possible.

Several previous studies have observed elevated frontal encoding activity in pathological aging and suggested that it may serve a compensatory role (Miller et al., 2008; Browndyke et al., 2013). In contrast, the present data indicate that atypical frontal activity is detrimental. Specifically, right VLPFC activity was mainly seen during retrieval in younger age and normal aging. In contrast, for the dropouts, right VLPFC activation was seen during both encoding and retrieval, with no or weak modulation of activity between task states. Although causality is difficult to fully ascertain given the slow sampling rate of fMRI we also attempted to distinguish potential temporal ordering of the signals. In younger age and normal aging the DCM and lag analyses converged to suggest top-down influences of the right VLPFC on the hippocampus during retrieval only, whereas for dropouts the pattern of modulation was similar during encoding and retrieval. It should be stressed that the connectivity analyses revealed elevated connectivity also during the control task for dropouts, and the lag analysis did not support any difference in the ordering of PFC or hippocampus activation in the dropout group. These findings suggest disturbed connectivity more generally for the dropouts. However, the DCM analyses provided support for directionality of top-down signals using a single, but possibly more efficient, analytical framework by showing stronger connectivity during encoding and retrieval than during the control task for the dropouts. Also, the atypical right VLPFC to hippocampus connectivity at encoding was significantly higher for older dropouts than remainers.

Collectively, in young adults and normal aging, our findings support a role of frontal cortex in top-down biasing of hippocampal computations during memory encoding and retrieval. We have stressed the importance of the *right* VLPFC for episodic retrieval, which is consistent with previous findings (Lepage et al., 2000; Habib et al., 2003; Salami et al., 2010) and more generally with the view that VLPFC-MTL interactions supports memory retrieval *via* pattern completion (Simons and Spiers, 2003). It should be noted that connectivity between VLPFC and the hippocampus, while for model simplicity was specified as a direct connection in our DCM analysis, in reality implicates additional cortical and subcortical regions (e.g., Eichenbaum, 2017). In pathological aging, here defined on basis of longitudinal study drop-out, dysfunctional hippocampal hyper-activity at encoding seems to reflect disturbed top-down signaling, notably right VLPFC involvement also during encoding, possibly resulting in triggering of retrieval/pattern completion in addition to encoding/pattern separation. This view is in line with findings of dynamic state-switching or multiplexing of neuronal ensembles within a single task (Gilbert and Sigman, 2007).

Disturbed state signaling could contribute to unbalance between pattern separation and completion and hippocampal hyper-activity also in other conditions, such as schizophrenia (Weiss and Heckers, 2001), and the basis for disturbed state influences may vary among conditions. In pathological aging, one possible mechanism is cholinergic loss (Schliebs and Arendt, 2011). Cholinergic loss can negatively influence encoding and retrieval state signals from prefrontal regions to the hippocampus and induce a shift towards retrieval and pattern-completion (Hasselmo and McGaughy, 2004; Bentley et al., 2011). Here it should be stressed that local modulation of cholinergic action in the hippocampus has been related to switching between learning and recall states (Hasselmo et al., 1995), and more generally that influential accounts of hippocampal hyper-activity in aging and shifts between encoding and retrieval have been put forward on basis of local hippocampal alterations and modulation of hippocampal processing by the input (see Wilson et al., 2006; Leal and Yassa, 2018). Therefore, the current top-down account should be seen as providing a complementary rather than a competing perspective on hippocampal hyper-activity and computational flexibility.

Finally, it must be acknowledged that our characterization of the task blocks in terms of encoding *or* retrieval likely is an over-simplification as tasks are not process pure. Thus, likely, intentional or incidental retrieval processes were operating also during encoding for all, including younger, participants. Relatedly, elevated right frontal activity during encoding for dropouts cannot uniquely be seen as indexing retrieval and hippocampal pattern-completion processes. One potential alternative account could be increased “system noise” and less distinct computations in pathological aging (Li et al., 2001).

In conclusion, while our interpretation of the present pattern of results in terms of top-down modulation of hippocampal computation awaits replication, it extends previous suggestions that bottom-up input serves to bias the hippocampus towards pattern separation or pattern completion (Guzowski et al., 2004). A key role of frontal state signals is consistent with findings of attentional effects on hippocampal processing during encoding and retrieval (Muzzio et al., 2009; Aly and Turk-Browne, 2016), and more generally with empirical and theoretical arguments that a region’s function is partly determined by its pattern of interactions with other regions (Bressler and McIntosh, 2007). Here, in the context of a general pattern of age-related anterior hippocampal hypo-activation during encoding, our findings suggest that disturbed fronto-hippocampal interactions contribute to paradoxically high anterior hippocampal activity in older dropouts at risk of pathology. Thus, whether high hippocampal activity is related to good memory performance (as in younger age and normal aging) or poor memory performance (as in pathological aging) can be decoded from distal patterns of regional activity (i.e., modulation of prefrontal regional activity between encoding and retrieval states). In future studies, analyses of functional interactions of the hippocampus with regions that do *not* display activation differences between conditions, but still interact differently with the hippocampus during different task and rest states (see McIntosh et al., 1997; Di and Biswal, 2018), might yield additional information on how efficient mnemonic functioning emerges from network interactions in the brain.

## Ethics Statement

The research was approved by the local ethics board at Umeå University, and all participants provided written informed consent and were compensated monetarily for their participation.

## Author Contributions

LN designed the study. LN, MA, AL, AS, and AW contributed to the data analyses and corresponding interpretations. All authors contributed in revising the article and approved the final version of the manuscript.

## Conflict of Interest

The authors declare that the research was conducted in the absence of any commercial or financial relationships that could be construed as a potential conflict of interest.

## Footnotes

^1^http://www.fil.ion.ucl.ac.uk/spm
